# Effects of cultivating biotech maize GG2 and glyphosate treatment on the rhizospheric microbial community structure

**DOI:** 10.1007/s42994-025-00205-8

**Published:** 2025-03-12

**Authors:** Yinxiao Wang, Yihe Hao, Shengyan Li, Ning Wen, Mingyuan Yin, Zhihong Lang

**Affiliations:** 1 National Nanfan Research Institute, Chinese Academy of Agricultural Sciences, Sanya, 572000 China; 2 Biotechnology Research Institute, Chinese Academy of Agricultural Sciences, Beijing, 100081 China

**Keywords:** Biotech maize, Glyphosate, Bacterial and fungal community composition, Risk assessment, Illumina NovaSeq PE250 sequencing

## Abstract

In environmental biosafety assessments of glyphosate-tolerant crops, it is essential to evaluate the effects of cultivating these crops and applying glyphosate on the microbial community in the rhizosphere soil, which play a critical role in maintaining soil health, plant growth, and crop productivity. Maize (*Zea mays*) line GG2 was previously generated by transforming wild-type maize with the *gat* and *gr79-epsps* genes, endowing GG2 with both active and passive resistance to glyphosate. However, the ecological risk of introducing these two new glyphosate-tolerance genes into maize, as well as glyphosate treatment, to rhizosphere microorganisms remain unclear. In this study, we used high-throughput sequencing to analyze the diversity and composition of the bacterial and fungal communities in the rhizosphere soil around biotech maize GG2, with (GG2-H) and without glyphosate treatment (GG2-N), compared with the near-isogenic, non-biotech maize line ZD958 at seven stages of growth. The structure and diversity of the bacterial and fungal communities of GG2-H were similar to those of ZD958, whereas glyphosate treatment had temporary effects on bacterial and fungal diversity and richness. The differences in the bacterial and fungal communities were associated with changes in soil properties such as pH, available phosphorus and organic matter, and seasonal changes. These factors, rather than maize lines, made the greatest contributions to the shifts in bacterial and fungal community structure. This study provides a comprehensive analysis of the effects of biotech crop cultivation, glyphosate treatment, soil physicochemical properties of soil, and maize growth stages on soil microbial communities, offering valuable insights for the large-scale adoption of biotech crops in China.

**Supplementary Information:**

The online version contains supplementary material available at 10.1007/s42994-025-00205-8.

## Introduction

Since biotech crops were first commercialized in 1996 in the United States, these crops have been rapidly adopted worldwide due to their high yield and stress resistance. The dominant trait of biotech crops has consistently been herbicide tolerance in soybean (*Glycine max*), canola, maize (*Zea mays*), alfalfa, and cotton (*Gossypium hirsutum*). In 2019, the cultivation area of herbicide-tolerant crops was 81.5 million hectares, accounting for 43% of the total Biotech crops (ISAAA 2019). Since 2023, the Ministry of Agriculture and Rural Affairs of China has approved 81 biotech maize and soybean varieties, including 64 maize varieties and 17 soybean varieties with the predominant traits being glyphosate (*N*-(phosphonomethyl)glycine) tolerance or combined glyphosate tolerance and insect resistance. In light of the rapid development of biotech crops with glyphosate tolerance, more attention should be paid to the potential risks of cultivating biotech crops and applying glyphosate.

Glyphosate is one of the most widely used herbicides in traditional agriculture due to its high efficiency and low toxicity. With the rapid development of biotech crops, the scope of glyphosate application has further expanded. It is important to examine the effects of glyphosate-tolerant crops and glyphosate application on the rhizosphere microbial community due to its critical roles in driving biogeochemical processes and controlling pathogens, ultimately enabling agricultural ecosystems to function and provide maximum value to humans. After glyphosate is absorbed by plants through the cuticle and/or stomata, its physicochemical properties cause it to be preferably distributed toward young roots, along with photoassimilates. The glyphosate is then excreted via root exudates into the rhizosphere soil, where it can affect microorganisms (Laitinen et al. 2007; Smedbol et al. 2019). Compared to bulk soil, rhizosphere soil occupies a narrow, dynamic zone in which complex interactions occur between root exudates and the soil microbiome (Coskun et al. 2017). The abundance of bacteria and fungi in the soil microbiome is much higher than that of other protists, and these organisms usually dominate the biomass and diversity of soil microorganisms (Bardgett and van der Putten 2014; Fierer 2017). Thus, the bacterial and fungal community structure in the rhizosphere soil can be used as an early, sensitive indicator for assessing the effects of growing glyphosate-resistant crops and treating plants with glyphosate on soil ecology (Kepler et al. 2020).

Many efforts have been made to assess the biosafety of applying glyphosate or cultivating biotech crops in terms of the effects on the soil microbial community (Van Bruggen et al. 2018; Kepler et al. 2020; Muskus et al. 2022; Shi et al. 2024). However, there is still much controversy about the effects of these practices on microbial communities and their activities in the soil. Glyphosate and its major metabolite aminomethylphosphonic acid (AMPA) can stimulate microbial activity. A few studies have looked at whether these compounds have toxic effects on soil microorganisms (Araujo et al. 2003; Aslam et al. 2023). For example, glyphosate treatment (applied at recommended or lower dosages) negatively affected the populations of microorganisms that promote plant growth, such as *Burkholderia* spp., *Pseudomonas* spp., arbuscular mycorrhizal fungi, and nitrogen-fixing rhizobial species (Zobiole et al. 2011; Arango et al. 2014; Druille et al. 2015). These treatments resulted in reduced plant growth and/or changes in grassland vegetation cover and composition (Arango et al. 2014; Druille et al. 2015). The microbial communities of soil subjected to short-term glyphosate treatment appeared to recover to a state similar to that of communities in untreated soil, with only minor or no effect on global microbial structure, biomass, or activity, likely due to the great diversity and compensatory activities of microorganisms in soil (Arango et al. 2014; Wolmarans and Swart 2014; Allegrini et al. 2015; Zabaloy et al. 2016). Planting glyphosate-tolerant maize line CC-2 harboring a modified *cp4-epsps* had no significant effects on the structure or diversity of the bacterial or fungal communities (Zhou et al. 2020). The insect-resistant maize line HGK60 had temporary effects on soil microorganisms during the growth cycle studied, however, the microorganism populations were restored after one cycle of plant cultivation (Wang et al. 2022). In general, the ecological effects of glyphosate or glyphosate-resistant crops on soil bacteria or fungi are far from conclusive; they may be positive, neutral, or detrimental. Moreover, the potential interactions of glyphosate-resistant plants, glyphosate treatment, and soil microorganisms have not been examined comprehensively. It is essential to comprehensively investigate the potential environmental risks of glyphosate and glyphosate-resistant crops on the soil microbial community. Furthermore, these issues should be analyzed on a case-by-case basis.

The *gat* and *gr79-epsps* genes were initially isolated from soil microorganisms growing in extremely glyphosate-polluted soil. These genes participate in different glyphosate-resistance mechanisms (Dun et al. 2006; Jin et al. 2007). Glyphosate achieves weed control by disrupting the activity of the enzyme 5-enolpyruvylshikimate-3-phosphate synthase (EPSPS) in the shikimate pathway. GR79-EPSPS exhibits high catalytic activity in the shikimate pathway but a low affinity for glyphosate, pointing to its great potential for engineering glyphosate-tolerant plants. Glyphosate-*N*-acetyltransferase (GAT) acetylates glyphosate to produce *N*‐acetylglyphosate, which can be further metabolized to the nonphytotoxic compounds *N*‐acetyl‐aminomethyl phosphonic acid (*N*‐acetyl‐AMPA) and AMPA (EFSA 2009). Co‐expression of codon-optimized forms of *gat* and *gr79-epsps* is an effective strategy for developing high glyphosate–resistant, low‐glyphosate‐residue crops that has been successfully used in upland cotton, maize, and alfalfa (Liang et al. 2017; Li et al. 2023; Meng et al. 2023). The transgenic maize line GG2 harboring *gat* and *gr79-epsps* is currently undergoing environmental assessment and food/feed biosafety assessment prior to its commercialization. Compared to previous glyphosate-tolerant crops with only one glyphosate-insensitive *epsps* gene, such as maize line CC-2, the environmental risks of GG2 maize, with both active and passive glyphosate resistance, require further assessment.

In this study, we examined the changes in the diversity and composition of the bacterial and fungal communities in the rhizosphere soil around GG2 treated with glyphosate (GG2-H), GG2 without glyphosate treatment (GG2-N), and the near-isogenic non-transgenic maize line ZD958 without glyphosate treatment (ZD-N) at seven stages of growth. We also comprehensively evaluated the ecological risks of cultivating GG2 and applying glyphosate to plants at the 4-leaf seedling stage on microbial community composition and diversity. In addition to providing useful information about the impact of growing glyphosate-resistant transgenic maize and glyphosate treatment on the soil microbiome, our findings provide a theoretical basis for improving the safety assessment approach for new herbicide-resistant maize lines in China.

## Results

### Physical and chemical characteristics of soil containing ZD958 and GG2 at different developmental stages

The physicochemical properties of soil are important attributes of the solid phase of soil; they are the main determinants of the availability of plant nutrients, which also influences the types and biomass of microorganisms present in soil and their functional identities. Therefore, soil nutrients, the microbial community, and plants work together to help maintain ecosystem function. We measured the nitrogen (N), potassium (K), and phosphorus (P) levels and other indices from rhizosphere soil samples from maize at different developmental stages (Table 1). There was no significant difference in alkali-hydro nitrogen (AN) content among soil samples during all developmental stages, and the contents of available potassium (AK), available phosphorus (AP), and organic matter (OM) and the pH value were similar among the GG2-H, GG2-N, and ZD-N samples at most stages of development. Differences in the physicochemical properties of the soil among GG2-H, GG2-N, and ZD-N were only observed in the early stages of maize growth. For example, the AK contents of ZD-N were different from those of GG2-H at the seedling stage with three leaves (SSV3), and the AP contents of ZD-N were higher than those of GG2-N at the SSV3 and heading stage (HS). The physical and chemical characteristics of the soil, including AN, AK, AP, OM, and pH value, were similar among GG2-H, GG2-N, and ZD-N at the dough stage (DS) and post-harvest stage (PHS).

**Table 1:** Physical and chemical characteristics of soils from different groups

Stage	Group	AN	AK	AP	OM	pH value
Preplant stage	GG2-H	5.04 ± 2.93^a^	98.10 ± 1.32^a^	6.15 ± 0.17^a^	5.94 ± 0.08^a^	8.78 ± 0.06^a^
	GG2-N	3.02 ± 0.38^a^	97.72 ± 2.14^ab^	6.08 ± 0.33^a^	6.60 ± 0.42^a^	8.77 ± 0.03^a^
	ZD958	3.97 ± 1.43^a^	95.35 ± 0.07^b^	8.00 ± 0.43^b^	5.55 ± 0.79^a^	8.74 ± 0.02^a^
Seedling stage (V3)	GG2-H	3.78 ± 1.31^a^	119.57 ± 10.56^a^	12.07 ± 4.65^ab^	8.79 ± 1.23^a^	8.24 ± 0.03^a^
	GG2-N	3.40 ± 1.00^a^	127.43 ± 9.08^ab^	10.88 ± 1.06^a^	8.08 ± 1.15^a^	8.23 ± 0.12^a^
	ZD958	3.78 ± 0.38^a^	149.51 ± 13.87^b^	14.22 ± 1.27^b^	9.54 ± 0.98^a^	8.23 ± 0.03^a^
Seedling stage (V5)	GG2-H	4.03 ± 0.79^a^	87.95 ± 12.16^a^	11.10 ± 4.60^a^	8.25 ± 1.48^a^	8.63 ± 0.02^a^
	GG2-N	3.27 ± 0.79^a^	100.35 ± 20.63^a^	10.57 ± 3.05^a^	11.72 ± 1.14^b^	8.52 ± 0.06^b^
	ZD958	3.40 ± 1.00^a^	88.21 ± 19.54^a^	11.70 ± 1.70^a^	9.45 ± 0.09^a^	8.55 ± 0.09^ab^
Heading stage	GG2-H	4.79 ± 1.79^a^	83.44 ± 13.62^a^	10.23 ± 3.00^ab^	8.80 ± 1.79^a^	8.52 ± 0.06^a^
	GG2-N	4.53 ± 0.76^a^	80.15 ± 10.77^a^	11.85 ± 1.41^a^	9.07 ± 2.46^a^	8.51 ± 0.01^a^
	ZD958	5.54 ± 2.65^a^	88.05 ± 17.01^a^	16.00 ± 2.13^b^	8.19 ± 0.78^a^	8.54 ± 0.03^a^
Silking stage	GG2-H	3.78 ± 2.30^a^	69.53 ± 8.29^a^	13.78 ± 5.78^a^	7.74 ± 1.14^a^	8.57 ± 0.03^a^
	GG2-N	4.79 ± 2.43^a^	67.39 ± 9.43^a^	12.00 ± 2.97^a^	9.51 ± 1.13^a^	8.53 ± 0.02^ab^
	ZD958	3.40 ± 0.38^a^	74.10 ± 7.78^a^	18.98 ± 5.99^a^	8.02 ± 0.76^a^	8.44 ± 0.06^b^
Dough stage	GG2-H	3.90 ± 1.43^a^	127.86 ± 31.92^a^	15.55 ± 3.72^a^	8.07 ± 1.10^a^	8.54 ± 0.03^a^
	GG2-N	3.78 ± 0.38^a^	108.86 ± 16.69^a^	10.43 ± 2.11^a^	8.34 ± 0.66^a^	8.56 ± 0.03^a^
	ZD958	5.16 ± 0.79^a^	125.05 ± 26.18^a^	12.70 ± 2.55^a^	8.69 ± 0.92^a^	8.43 ± 0.08^a^
Post-harvest stage	GG2-H	4.41 ± 0.58^a^	149.15 ± 34.35^a^	13.78 ± 3.25^a^	7.73 ± 0.79^a^	8.66 ± 0.02^a^
	GG2-N	3.53 ± 0.58^a^	161.12 ± 16.38^a^	15.12 ± 2.06^a^	8.57 ± 0.18^a^	8.56 ± 0.10^a^
	ZD958	3.40 ± 0.38^a^	140.05 ± 31.44^a^	15.47 ± 1.89^a^	8.01 ± 0.63^a^	8.64 ± 0.05^a^

All values are given as mean ± SD, and different letters indicate a significant difference between GG2-H, GG2-N, and ZD-N at *P* < 0.05 according to one-way ANOVA (*n* = 3)

*AN* alkali-hydro nitrogen, *AK* available potassium, *OM* organic matter, *AP* available phosphorus, *GG2-H* GG2 treated with glyphosate at the seedling stage with four leaves, *GG2-N* GG2 without glyphosate treatment, *ZD-N* ZD958 without glyphosate treatment

### Richness and diversity of the rhizosphere bacterial and fungal communities

**Fig. 1:**
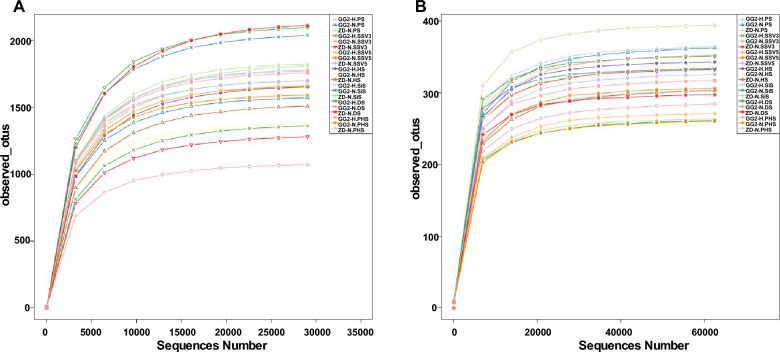
Rarefaction analysis. Rarefaction curve of the operational taxonomic units (OTUs) obtained from the V3V4 region of the 16S rRNA gene (**A**) and the ITS of the 18S rRNA gene and 5.8S rRNA gene (**B**), based on amplicon sequencing of the rhizosphere soils of GG2 and ZD958. *GG2-H* GG2 treated with glyphosate at the seedling stage with four leaves, *GG2-N* GG2 without glyphosate treatment, *ZD-N* ZD958 without glyphosate treatment, *PS* preplant stage, *SSV3* seedling stage with three leaves, *SSV5* seedling stage with five leaves, *HS* heading stage, *SiS* silking stage, *DS* dough stage, *PHS* post-harvest stage

We amplified the V3V4 region of the 16S rRNA gene and the ITS of the 18S rRNA and 5.8S rRNA genes from rhizosphere soil samples and sequenced them on an Illumina NovaSeq PE250 platform to determine the compositions of the bacterial and fungal communities, respectively. The rarefaction curves of operational taxonomic units (OTUs) showed no significant differences between GG2 and ZD958, and the data were sufficient for revealing differences in the bacterial and fungal communities (if any) between the different lines as well as different glyphosate treatments (Fig. 1). For the 16S rRNA gene, 28,340 OTUs were detected at 97% similarity without singletons. As shown in the Venn diagram (Fig. S1A), 14,639, 13,342, and 15,088 OTUs were detected in the rhizosphere soil of GG2-H, GG2-N, and ZD-N, respectively. Of these OTUs, 4924 OTUs were shared among all groups, 1149 were shared at all growth stages, 3149 were only observed at PS, 2056 at SSV3, 2594 at SSV5, 2440 at HS, 2389 at SiS, 2625 at DS, and 2430 at PHS, indicating that the number of OTUs varied at different developmental stages (Fig. S1B). For the ITS of the rRNA genes, 4388 OTUs were detected, including 2134 OTUs that were observed in GG2-H, 2296 in GG2-N, and 2202 in ZD-N, with 735 OTUs shared among all groups (Fig. S1C). Finally, we detected 503, 571, 689, 690, 743, 731, and 672 OTUs at PS, SSV3, SSV5, HS, SiS, DS, and PHS, respectively (Fig. S1D). These results indicate that the number of OTUs increased throughout the entire maize lifecycle.

We observed significant differences in bacterial and fungal richness and diversity for the OTUs, as revealed by measuring the Chao1, Dominance, Simpson, and Shannon diversity indices at different stages of plant growth (Table 2). Analysis of richness estimators, including the number of OTUs and the Chao1 index, indicated that there was no significant difference in the total number of bacterial and fungal species in the rhizosphere between GG2 and ZD958 or between glyphosate treatment and no treatment during all growth periods. There were also no significant differences in the bacterial or fungal diversity indices (Dominance, Simpson, and Shannon indices) during most growth periods, except for SSV5. Among bacterial diversity indices, the dominance and Shannon diversity indices of GG2-H and GG2-N were significantly different from those of ZD-N, suggesting that the two exogenous genes (*gr79-epsps* and *gat*) in GG2 affected the diversity of the bacterial communities in rhizosphere soil. Notably, there were no significant differences in diversity indices between GG2 and ZD958 at later stages of plant growth, particularly at the dough stage and post-harvest stage, and the Simpson index of GG2 was similar to that of ZD958 at SSV5. Thus, the differences in bacterial diversity indices between GG2 and ZD958 might have been temporary and random, and the bacterial richness and diversity of the rhizosphere soil were similar between biotech maize line GG2 and its near-isogenic non-transgenic maize line ZD958. The dominance, Simpson, and Shannon diversity indices of GG2-H were different from those of GG2-N and ZD-N. These results indicate that treating GG2 with glyphosate had a temporary effect on the fungal diversity of rhizosphere soil and that the integration of *gat* and *gr79-epsps* into GG2 had no impact on the diversity of the fungal communities.

**Table 2:** Diversity indices of rhizosphere bacteria and fungi in different groups

Category	Stage	Group	OTUs	Chao1	Dominance	Shannon	Simpson
Bacteria	Preplant stage	GG2-H	1772.33 ± 172.76^a^	1784.79 ± 182.96^a^	0.005 ± 0.003^a^	9.67 ± 0.27^a^	0.995 ± 0.003^a^
		GG2-N	1572.33 ± 70.74^a^	1586.19 ± 66.16^a^	0.008 ± 0.006^a^	9.21 ± 0.41^a^	0.992 ± 0.006^a^
		ZD-N	1825.00 ± 337.76^a^	1841.72 ± 347.82^a^	0.013 ± 0.007^a^	9.35 ± 0.27^a^	0.986 ± 0.007^a^
	Seedling stage (V3)	GG2-H	1362.67 ± 248.15^a^	1380.03 ± 251.43^a^	0.012 ± 0.003^a^	8.53 ± 0.45^a^	0.988 ± 0.003^a^
		GG2-N	1071.33 ± 229.32^a^	1086.89 ± 240.25^a^	0.020 ± 0.006^a^	7.92 ± 0.49^a^	0.980 ± 0.006^a^
		ZD-N	1280.33 ± 340.21^a^	1303.10 ± 329.75^a^	0.019 ± 0.012^a^	8.25 ± 0.80^a^	0.981 ± 0.012^a^
	Seedling stage (V5)	GG2-H	1699.00 ± 52.60^a^	1712.19 ± 54.57^a^	0.004 ± 0.002^a^	9.65 ± 0.19^a^	0.996 ± 0.002^a^
		GG2-N	1658.00 ± 264.55^a^	1678.58 ± 276.99^a^	0.004 ± 0.001^a^	9.51 ± 0.24^a^	0.996 ± 0.001^a^
		ZD-N	1700.00 ± 269.29^a^	1708.00 ± 270.96^a^	0.014 ± 0.001^b^	9.15 ± 0.28^a^	0.986 ± 0.001^b^
	Heading stage	GG2-H	1652.67 ± 491.33^a^	1669.37 ± 501.96^a^	0.011 ± 0.006^a^	9.11 ± 0.48^a^	0.989 ± 0.006^a^
		GG2-N	1377.00 ± 188.18^a^	1393.10 ± 204.84^a^	0.019 ± 0.014^a^	8.76 ± 0.65^a^	0.981 ± 0.014^a^
		ZD-N	1509.33 ± 274.05^a^	1523.24 ± 282.48^a^	0.021 ± 0.009^a^	8.51 ± 0.43^a^	0.979 ± 0.009^a^
	Silking stage	GG2-H	1700.33 ± 262.98^a^	1713.58 ± 273.41^a^	0.003 ± 0.001^a^	9.73 ± 0.25^a^	0.997 ± 0.001^a^
		GG2-N	2037.00 ± 95.97^a^	2063.77 ± 88.25^a^	0.003 ± 0.001^a^	10.04 ± 0.04^a^	0.997 ± 0.001^a^
		ZD-N	1810.67 ± 70.23^a^	1835.80 ± 72.43^a^	0.004 ± 0.001^a^	9.57 ± 0.10^a^	0.996 ± 0.001^a^
	Dough stage	GG2-H	2096.00 ± 149.64^a^	2119.55 ± 152.10^a^	0.003 ± 0.000^a^	10.06 ± 0.07^a^	0.997 ± 0.000^a^
		GG2-N	1758.33 ± 319.99^a^	1779.43 ± 333.25^a^	0.008 ± 0.008^a^	9.55 ± 0.59^a^	0.992 ± 0.008^a^
		ZD-N	2111.00 ± 119.25^a^	2131.29 ± 114.77^a^	0.011 ± 0.010^a^	9.65 ± 0.31^a^	0.989 ± 0.010^a^
	Post-harvest stage	GG2-H	1665.00 ± 303.35^a^	1686.29 ± 316.25^a^	0.009 ± 0.003^a^	9.29 ± 0.45^a^	0.991 ± 0.003^a^
		GG2-N	1592.00 ± 220.58^a^	1600.71 ± 229.93^a^	0.011 ± 0.004^a^	9.29 ± 0.04^a^	0.989 ± 0.004^a^
		ZD-N	1780.33 ± 230.89^a^	1795.85 ± 240.09^a^	0.010 ± 0.007^a^	9.44 ± 0.26^a^	0.990 ± 0.007^a^
Fungi	Preplant stage	GG2-H	303.67 ± 59.87^a^	304.46 ± 59.98^a^	0.062 ± 0.058^a^	5.69 ± 0.71^a^	0.938 ± 0.058^a^
		GG2-N	362.67 ± 67.99^a^	364.94 ± 68.99^a^	0.067 ± 0.032^a^	5.28 ± 0.81^a^	0.933 ± 0.032^a^
		ZD-N	263.67 ± 75.63^a^	263.98 ± 75.67^a^	0.089 ± 0.081^a^	5.21 ± 0.74^a^	0.911 ± 0.081^a^
	Seedling stage (V3)	GG2-H	261.33 ± 48.01^a^	263.48 ± 46.92^a^	0.114 ± 0.024^a^	4.88 ± 0.24^a^	0.886 ± 0.024^a^
		GG2-N	284.67 ± 41.79^a^	285.65 ± 42.16^a^	0.070 ± 0.056^a^	5.30 ± 0.76^a^	0.930 ± 0.056^a^
		ZD-N	332.33 ± 6.66^a^	332.73 ± 6.31^a^	0.076 ± 0.012^a^	5.30 ± 0.28^a^	0.924 ± 0.012^a^
	Seedling stage (V5)	GG2-H	351.67 ± 28.57^a^	352.67 ± 28.90^a^	0.026 ± 0.005^a^	6.34 ± 0.12^a^	0.974 ± 0.005^a^
		GG2-N	306.00 ± 31.18^a^	306.77 ± 31.81^a^	0.089 ± 0.06^ab^	5.14 ± 0.50^b^	0.911 ± 0.063^ab^
		ZD-N	325.67 ± 23.18^a^	326.63 ± 22.81^a^	0.057 ± 0.015^b^	5.59 ± 0.19^b^	0.943 ± 0.015^b^
	Heading stage	GG2-H	343.000 ± 57.30^a^	343.24 ± 57.31^a^	0.038 ± 0.003^a^	6.01 ± 0.33^a^	0.962 ± 0.003^a^
		GG2-N	335.33 ± 35.16^a^	335.53 ± 34.95^a^	0.037 ± 0.005^a^	5.96 ± 0.13^a^	0.963 ± 0.005^a^
		ZD-N	303.33 ± 27.93^a^	305.10 ± 29.66^a^	0.062 ± 0.022^a^	5.44 ± 0.38^a^	0.938 ± 0.022^a^
	Silking stage	GG2-H	364.333 ± 25.81^a^	364.94 ± 25.52^a^	0.064 ± 0.026^a^	5.64 ± 0.49^a^	0.936 ± 0.026^a^
		GG2-N	333.33 ± 47.54^a^	333.91 ± 47.18^a^	0.039 ± 0.019^a^	6.06 ± 0.39^a^	0.961 ± 0.019^a^
		ZD-N	393.67 ± 27.47^a^	394.32 ± 27.78^a^	0.093 ± 0.074^a^	5.35 ± 1.00^a^	0.907 ± 0.074^a^
	Dough stage	GG2-H	350.67 ± 30.35^a^	351.96 ± 31.16^a^	0.025 ± 0.005^a^	6.37 ± 0.27^a^	0.975 ± 0.005^a^
		GG2-N	317.00 ± 56.67^a^	317.67 ± 56.58^a^	0.058 ± 0.030^a^	5.69 ± 0.39^a^	0.942 ± 0.030^a^
		ZD-N	297.33 ± 49.00^a^	299.28 ± 47.51^a^	0.043 ± 0.014^a^	5.90 ± 0.29^a^	0.957 ± 0.014^a^
	Post-harvest stage	GG2-H	271.00 ± 53.33^a^	271.35 ± 53.17^a^	0.048 ± 0.027^a^	5.55 ± 0.78^a^	0.952 ± 0.027^a^
		GG2-N	260.33 ± 56.01^a^	262.51 ± 57.76^a^	0.042 ± 0.015^a^	5.70 ± 0.47^a^	0.958 ± 0.015^a^
		ZD-N	352.67 ± 73.19^a^	353.53 ± 72.86^a^	0.052 ± 0.015^a^	5.66 ± 0.14^a^	0.948 ± 0.015^a^

Note: OTUs: the number of operational taxonomic units; Chao1: the total number of species in the estimated community; Dominance, Shannon and Simpson: the diversity and evenness of species distribution in the community. GG2-H: GG2 treated with glyphosate at the seedling stage with four leaves. GG2-N: GG2 without glyphosate treatment. ZD-N: ZD958 without glyphosate treatment. All values are given as mean ± SD, and different letters indicate a significant difference between GG2-H, GG2-N, and ZD-N at *P* < 0.05 according to one-way ANOVA (*n* = 3)

### Bacterial and fungal community composition

**Fig. 2:**
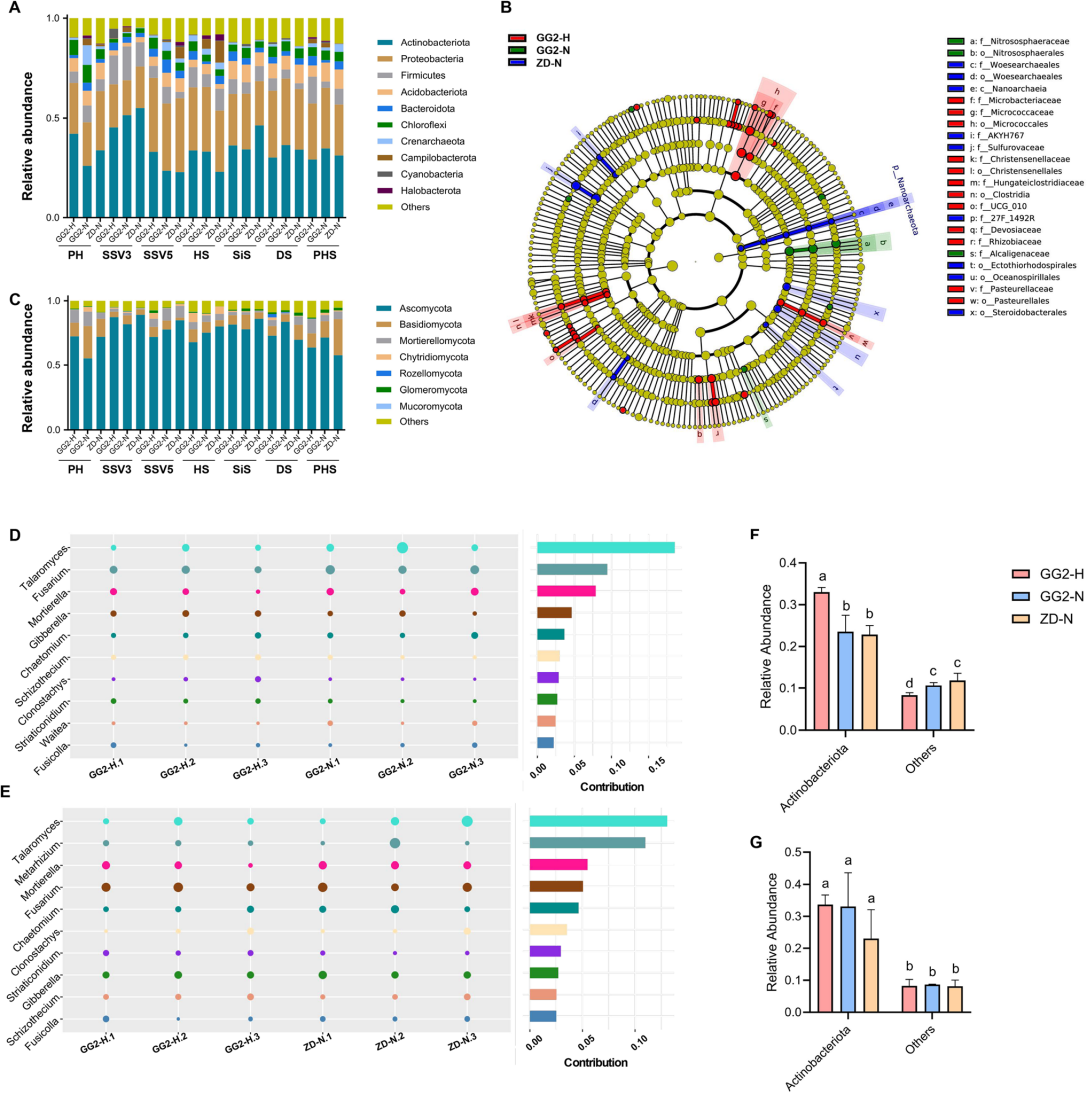
Bacterial and fungal community composition in the rhizosphere soils of GG2 and ZD958. **A** Relative abundances of the top ten bacterial phyla. **B** Relative abundances of Actinobacteriota and the other phyla (excluding the top ten phyla) at SSV5. **C** Relative abundances of Actinobacteriota and the other phyla (excluding the top ten phyla) at HS. **D** The bacterial biomarkers of GG2-H, GG2-N, and ZD-N at SSV5. **E** Relative abundances of the top ten fungal phyla. **F** SIMPER analysis of fungal community composition between GG2-H and GG2-N at SSV5. **G** SIMPER analysis of fungal community composition between GG2-H and ZD-N at SSV5. *GG2-H* GG2 treated with glyphosate at the seedling stage with four leaves, *GG2-N* GG2 without glyphosate treatment, *ZD-N* ZD958 without glyphosate treatment, *PS* preplant stage, *SSV3* seedling stage with three leaves, *SSV5* seedling stage with five leaves, *HS* heading stage, *SiS* silking stage, *DS* dough stage, *PHS* post-harvest stage. All values are given as mean ± SD, and different letters indicate a significant difference between GG2-H, GG2-N, and ZD-N at *P* < 0.05 according to one-way ANOVA (*n* = 3)

Phylogenetic classification of rhizosphere bacteria based on the V3V4 hypervariable sequences of the 16S rRNA gene and the ITS of 18S rRNA and 5.8S rRNA genes for all samples is shown in Fig. S2A. For the bacterial community, Actinobacteriota was the dominant phylum, with an average of 35.02% relative abundance, followed by Proteobacteria (28.42%), Firmicutes (7.90%), Acidobacteriota (6.24%), Chloroflexi (4.51%), Bacteroidota (3.30%), Crenarchaeota (2.04%), Campylobacterota (1.63%), Cyanobacteria (0.60), and Halobacterota (0.50%). These phyla accounted for > 90% of relative bacterial abundance (Fig. 2A, Table S1). Based on the finding that significant differences in bacterial diversity indices among the three treatments were only detected at SSV5, we analyzed the differences in bacterial community composition at the phylum level at SSV5 (Table S2). There were no significant differences in the relative abundance of the top ten rhizosphere bacteria phyla between GG2-N and ZD-N at SSV5 (Table S2). The relative abundance of Actinobacteriota in the rhizosphere soil of GG2-H was significantly higher than those of GG2-N and ZD-N, and the relative abundance of other phyla was significantly lower than those of GG2-N and ZD-N at SSV5 (Fig. 2F), whereas no significant difference was detected at HS (Fig. 2G). It appears that treating GG2 with glyphosate had a temporary effect on the bacterial community composition of rhizosphere soil at the phylum level.

To further clarify the effect of glyphosate treatment on bacterial composition at the genus level, we performed Linear discriminant analysis effect size (LEFSE, Wilcoxon rank sum test, *P* < 0.05, LDA score > 4) at SSV5 (Fig. 2B). The relative abundances of the genera *Pseudarthrobacter, Umezawaea, Salinispora, Rothia, Agromyces, Luedemannella, Salinispora, Dactylosporangium, Modestobacter*, and *Virgisporangium*, belonging to the Actinobacteriota phylum, were higher in the rhizosphere soil of GG2-H compared to GG2-N and ZD-N, while the relative abundances of *Woesearchaeales* and 27F_1492R genera, which are classified as Nanoarchaeota and Myxococcota, respectively, were higher in the rhizosphere soil of GG2-N or ZD-N than GG2-H. The changes in the relative abundances of these genera might have been related to the application of glyphosate.

Phylogenetic classification of rhizosphere fungi based on amplification of the ITS sequences of all samples is shown in Fig. S2B. Among the phyla (relative abundance > 1%), Ascomycota was the most dominant, with an average of relative abundance of 75.06%, followed by Basidiomycota (9.76%), Mortierellomycota (5.42%), Chytridiomycota (1.66%), and Glomeromycota (1.03%) (Fig. 2C, Table S3). There were no significant differences in the relative abundances of the four major rhizosphere fungal phyla between GG2 and ZD958 during almost all growth periods, except at SSV5 and HS (Table S4). At HS, the relative abundances of Ascomycota and Glomeromycota in the rhizosphere soil of GG2-H were significantly different from those of ZD-N. At SSV5, the relative abundances of Ascomycota, Basidiomycota, Chytridiomycota, and Glomeromycota in the rhizosphere soil of GG2-H were significantly different from those of GG2-N or ZD-N, while there was no significant difference between GG2-N and ZD-N. The relative abundances varied in a similar manner in the rhizospheres of both GG2-N and ZD-N at different growth stages, and the fungal community composition at the phylum level showed no significant difference between GG2-N and ZD-N.

To gain insight into the impact of glyphosate treatment on fungal composition at the genus level, we conducted SIMPER analysis. At SSV5, the top ten genera that contributed to the differences between GG2-H and GG2-N were *Talaromyces, Fusarium, Mortierella, Gibberella, Chaetomium, Schizothecium, Clonostachys, Striaticonidium, Waitea*, and *Fusicolla*. The top ten genera that contributed to the differences between GG2-H and ZD-N were *Talaromyces, Metarhizium, Mortierella, Fusarium, Chaetomium, Clonostachys, Striaticonidium, Gibberella, Schizothecium*, and *Fusicolla* (Fig. 2D, E). Therefore, the nine overlapping genera in these top ten groups, including *Talaromyces, Fusarium, Mortierella, Gibberella, Chaetomium, Schizothecium, Clonostachys, Striaticonidium*, and *Fusicolla*, might be the genera that are most strongly affected by the application of glyphosate.

Arbuscular mycorrhizal fungi (AMF) are a group of non-target microorganisms that can form mutualistic symbioses with the roots of most plant species, providing nutrients and enhancing plant disease resistance and stress tolerance (Steinkellner et al. 2012). AMF are more sensitive to changes in the physiology of the host plant than other soil microorganisms and could serve as key non-target microorganisms to be monitored when assessing the environmental impact of transgenic plants (Giovannetti et al. 2002). Thus, we focused on the effects of maize lines and glyphosate on AMF. Twelve genera belonging to Glomeromycota were detected in the rhizosphere soil, and *Glomus* and *Funneliformis* were the dominant genera of AMF. There were no significant differences in the relative abundances of any AMF genera between lines GG2 and ZD958 during any growth period, and between GG2 with or without glyphosate treatment which indicated that AMF were insensitive to glyphosate (Table S5).

### Effects of plant growth stages on the rhizosphere bacterial and fungal communities

**Fig. 3:**
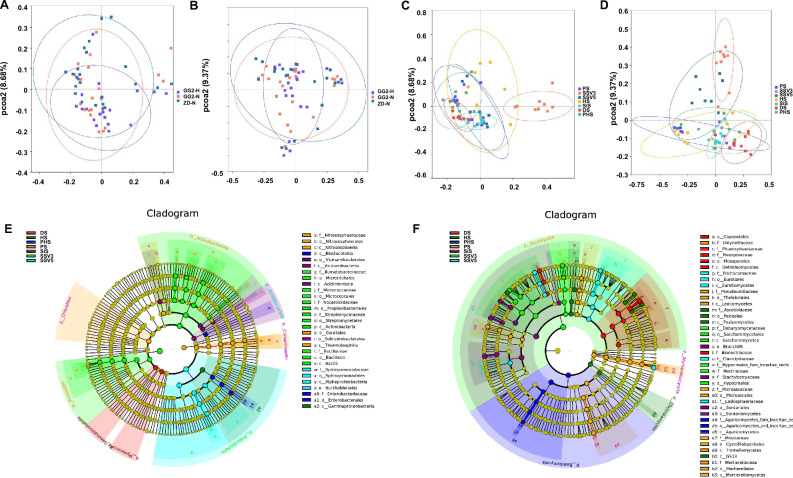
Effects of plant growth stages on the rhizosphere bacterial and fungal communities. **A**, **B** Principal Coordinates Analysis (PCoA) of bacterial (**A**) and fungal (**B**) community structure between maize lines. **C**, **D** PCoA of bacterial (**C**) and fungal (**D**) community structure of the rhizosphere of maize at different growth stages. **E**, **F** Results of linear discriminant analysis effect size (LEFSE) based on the 21 compartments of data to identify biomarkers of the bacterial (**E**) and fungal (**F**) communities in the rhizosphere of maize at different growth stages. *GG2-H* GG2 treated with glyphosate at the seedling stage with four leaves, *GG2-N* GG2 without glyphosate treatment, *ZD-N* ZD958 without glyphosate treatment, *PS* preplant stage, *SSV3* seedling stage with three leaves, *SSV5* seedling stage with five leaves, *HS* heading stage, *SiS* silking stage, *DS* dough stage, *PHS* post-harvest stage

We performed Principal Coordinates Analysis (PCoA) to examine the similarity of bacterial and fungal community compositions and to identify the differences in these communities between maize lines and growth stages (Fig. 3). Using maize line as an explanatory variable, we found no significant differences in the rhizosphere bacterial and fungal communities between GG2 and ZD958 (Fig. 3A, B). The confidence intervals of different growth stages were significantly different, and the bacterial and fungal community structure in the rhizosphere soil was marginally related to plant growth stages (Fig. 3C, D). The results of PERMANOVA confirmed the PCoA plot, showing that the effects of lines and growth stages on the bacterial and fungal community composition were consistent, as shown in Table 3. According to the ADONIS values, the effect of growth stages on bacterial and fungal community composition was greater than that of lines, likely due to changes in root exudation with plant maturity, as well as seasonal variation (Chaparro et al. 2014).

**Table 3:** Results of PERMANOVA of the association of bacterial and fungal community structure

Category	F. model	R^2^	Pr (> F)
Bacteria			
Lines	1.36	0.03	0.20
Growth stages	2.86	0.43	0.001
Fungi			
Lines	1.18	0.03	0.25
Growth stages	3.63	0.19	0.001

To identify biomarkers of the bacterial and fungal communities at different stages of plant growth, we employed Linear discriminant analysis effect size (LEFSE, Wilcoxon rank sum test, *P* < 0.05, LDA score > 4) based on the 42 compartments of data (Fig. 3E, F). The bacterium *Nitrososphaeraceae* and various fungi (*Tausonia, Cephalotrichum, Mortierella*, and *Pseudogymnoascus*) were enriched at PS. Various bacteria (*Nocardioides, Bacillus, Pseudarthrobacter*, CL500_29_marine_group, and *Streptomyces*) and fungi (*Sarocladium, Meyerozyma, Sarocladium*, and *Fusarium*) were enriched at SSV3. The bacterium *Sphingomonas* and several fungi (*Metarhizium, Talaromyces*, and *Mortierella*) were enriched at SSV5. *Fusarium, Alternaria*, and *Exserohilum* were the fungal community biomarkers at HS, and *Gibberella*, *Chaetomium*, and *Striaticonidium* were the fungal community biomarkers at SiS. Several fungi (*Clonostachys, Bipolaris*, and *Clonostachys*) were enriched at the dough stage, while *Escherichia_Shigella* and *Akenomyces* were the bacterial and fungal community biomarkers at the post-harvest stage, respectively. These results suggest that the differences in the bacterial and fungal communities were marked by seasonality, which is an environmental factor.

### Effects of soil indices on the rhizosphere bacterial and fungal communities

**Fig. 4:**
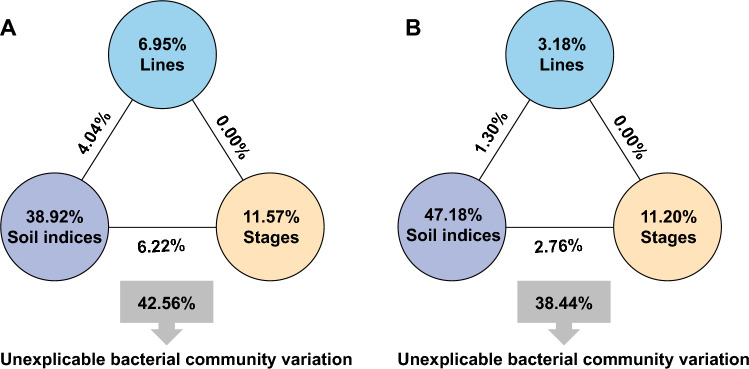
Effects of lines, stages, and soil indices on the bacterial and fungal communities of the rhizosphere. **A** Variance partitioning analysis (VPA) of the effects of lines, growth stages, soil indices, and the interactions among these factors on bacterial community structure; **B** VPA of the effects of lines, growth stages, soil indices, and the interactions among these factors on fungal community structure. Circles show the percentage of variation caused by each factor alone. The percentages of variation caused by interactions of two factors are shown on the horizontal lines. The unexplained variation is shown in rectangles below the figure

To gain insight into the impacts of the physical and chemical characteristics of soil on the bacterial and fungal communities, we performed variance partitioning analysis (VPA) to quantify the contribution values. Variations in the bacterial or fungal community structure were partitioned among soil indices, lines and growth stages, as well as the interactions among these variables. These variables explained 57.44% of the observed variation in bacterial composition and 61.56% of the observed variation in fungal composition, leaving 42.56% and 38.44% of the variation unexplained, respectively (Fig. 4A, B). Soil indices explained 38.92% and 47.18% of the variation in bacterial and fungal composition, while lines and growth stages accounted for 6.95% and 11.57% of the variation in bacterial composition and 3.18% and 11.20% of the variation in fungal composition, respectively. Thus, soil indices were the most important factors contributing to the shifts in the bacterial and fungal community structure.

**Fig. 5:**
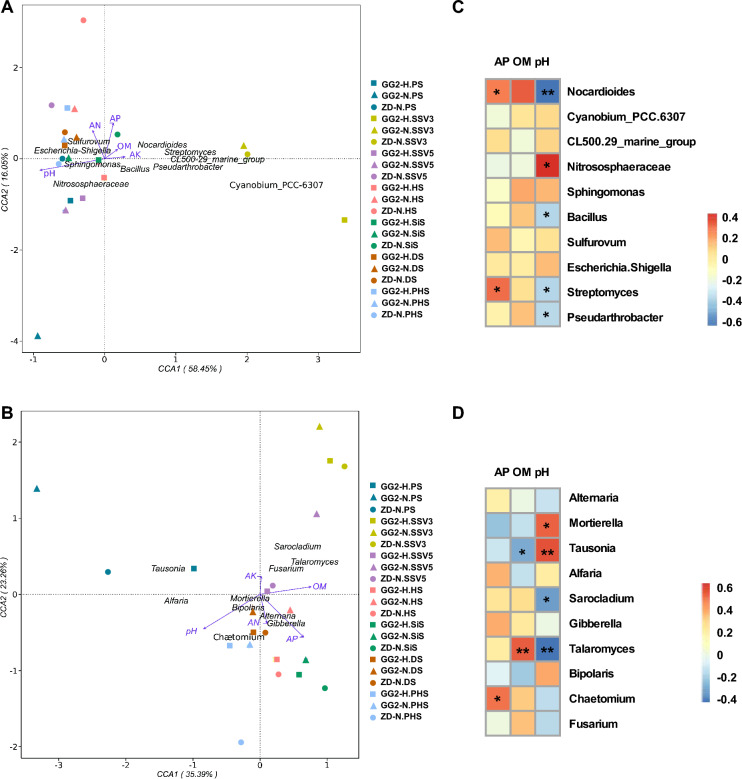
Effects of different soil characteristics on the bacterial and fungal communities. **A** Canonical Correlation Analysis (CCA) of the effects of different soil characteristics on the bacterial community. **B** CCA of the effects of different soil characteristics on the fungal community. **C** Spearman correlation analysis of the impact of soil characteristics on bacterial genera. **D** Spearman correlation analysis of the impact of soil characteristics on fungal genera. *GG2-H* GG2 treated with glyphosate at the seedling stage with four leaves, *GG2-N* GG2 without glyphosate treatment, *ZD-N* ZD958 without glyphosate treatment, *PS* preplant stage, *SSV3* seedling stage with three leaves, *SSV5* seedling stage with five leaves, *HS* heading stage, *SiS* silking stage, *DS* dough stage, *PHS* post-harvest stage

To further clarify the effects of different soil characteristics on the bacterial and fungal communities, we performed Canonical Correlation Analysis (CCA) and Spearman correlation analysis. pH and AP were the principal factors contributing to the shifts in bacterial community structure, while pH, OM, and AP were the major factors contributing to the variation in the fungal community (Fig. 5A, B). Moreover, pH was negatively correlated with OM and AP in terms of affecting the composition of bacteria and fungi. Further analysis showed that several bacterial genera (*Pseudarthrobacter, Streptomyces, Nocardioides*, and *Bacillus*) and fungal genera (*Talaromyces* and *Sarocladium*) were negatively correlated with pH, while the bacterial genus *Nitrososphaeraceae* and the fungal genera *Tausonia* and *Mortierella* were positively correlated with pH. The bacterial genera *Streptomyces* and *Nocardioides* and the fungal genus *Chaetomium* were positively correlated with AP. There was a significant positive correlation between the fungal genus *Talaromyces* and OM, while the fungal genus *Tausonia* was negatively correlated with OM (Fig. 5C, D). The bacterial and fungal genera mentioned above may be closely related to the physical and chemical characteristics of the soil and play vital roles in nutrient transformation, nutrient storage, and nutrient cycling.

## Discussion

Continuous monitoring throughout the growth period is required to verify whether transgenic plants have a persistent effect on the microbial community in the rhizosphere soil (Bai et al. 2019). In this study, we analyzed the bacterial and fungal communities in rhizosphere soil of GG2-H, GG2-N, and ZD-N at seven different stages of growth. The bacterial and fungal community composition of GG2-N was similar to that of ZD-N. Compared to maize lines, the plant growth stage was an important factor contributing to the shifts in bacterial and fungal community structure. These results are consistent with our previous findings on biotech maize line HGK60 (Wang et al. 2022). In that study, we analyzed the bacterial and fungal communities in rhizosphere soil of HGK60 and ZD958 at five different stages of growth. In the current study, to clarify the effect of seasons on the composition of bacteria and fungi in the rhizosphere soil, we used the rhizosphere soils of ZD958 as the control for HGK60 in 2021 and GG2 in 2022 (Fig. S3). Linear discriminant analysis effect size (LEFSE, Wilcoxon rank sum test, *P* < 0.05, LDA score > 2) showed that only a few bacterial and fungal genera were the biomarkers of the same growth stage in different years. Moreover, in our previous study of microbial community in the rhizosphere soil of HGK60 and ZD958, Proteobacteria and Ascomycota were the most dominant phyla of bacteria and fungi, whereas Actinobacteriota and Ascomycota were the most dominant phyla of bacteria and fungi in the rhizosphere soils of both GG2 and ZD958. Comprehensive comparative analysis suggested that the dominant phyla of the bacterial community in the rhizosphere soil of ZD958 varied in different years and that year was a more significant factor than plant growth stage in driving shifts in the bacterial community structure.

Microorganisms fulfil a wide range of functions in soil, such as the release of nutrients from minerals and OM (Gadd 2007; Uroz et al. 2009), nitrogen fixation (Vitousek et al. 2002), and soil aggregation (Oades and Waters 1991). Microorganism community is also affected by soil properties, such as the parent material, texture, pH, and oxygen availability (redox conditions). In the current study, soil indices explained 38.92% and 47.18% of the variations in bacterial and fungal composition, respectively, which are much higher than the contributions of lines and growth stages. In addition, pH, OM, and AP were the principal factors contributing to the shifts in bacterial and community structure. Moreover, several bacteria and fungi might be related to pH or OM, such as the genera *Pseudarthrobacter* and *Nocardioides*, belonging to Actinomycetes, which contribute to the degradation of pollutants (Ma et al. 2023; Naloka et al. 2014). These results provide a reference for further analysis of how pH or OM affects soil microbial composition.

Glyphosate, widely used as a herbicide in agriculture, has potential non-target effects on soil functions, plant health, and crop productivity, which have always been a concern (Van Bruggen et al. 2018). In this study, we detected subtle, temporary changes to the rhizosphere bacterial community composition following treatment with the herbicide glyphosate. The relative abundance of Actinobacteriota increased following glyphosate exposure, and the relative abundances of Ascomycota, Basidiomycota, Chytridiomycota, and Glomeromycota also changed in response to this treatment. These results are not consistent with the results of a previous study by Newman et al. (Newman et al. 2016). The authors showed that the relative abundance of Proteobacteria (particularly Gammaproteobacteria) increased after glyphosate treatment and that the relative abundance of Acidobacteria decreased in response to this treatment (Newman et al. 2016). These discrepancies may be attributed to the different physical and chemical properties of the soils used in these studies. Soil texture, mineralogy, pH, and OM have major effects on microbial community structure and the fate, decomposition, and sorption of glyphosate and its metabolites, such as AMPA (Ascolani et al. 2014; Chang et al. 2023). The sorption of glyphosate increases with increasing surface area of minerals in soil and with decreasing soil pH (Duke et al. 2012). In Newman’s study, the pH value of the soil was 6.9, while the pH values of our soil samples were > 8.0. We did not examine several soil types with variable levels of chemicals and different physical properties to determine the toxicity effects on microbial community members. An important issue to address is how the effects of glyphosate on microbial components should be assessed. We suggest that (1) More crops should be examined, which would help eliminate interference from the uniqueness of rhizosphere microorganisms in different crops; (2) Extensive research on long-term glyphosate treatment should be conducted to investigate shifts in the rhizosphere bacterial community at a finer level both taxonomically and functionally; (3) More research is needed on the adsorption rate and microbial toxicity of glyphosate or AMPA in different soil types.

## Materials and methods

### Plant and soil materials

Two maize lines, including the transgenic maize line GG2 and the near-isogenic non-transgenic maize line Zhengdan 958 (ZD958), were planted in Langfang, Hebei, China (116.71° E and 39.52° N), in the summer of 2022. This area has a typical temperate continental monsoon climate with an average annual precipitation of 555.3 mm and temperature of approximately 11.9 °C. The total area of this experimental field was 900 m^2^, which was divided into 9 plots of 100 m^2^ with 3 replicate plots each for GG2-H treated with glyphosate at the seedling stage (four leaves), GG2-N without glyphosate treatment, and ZD-N without glyphosate treatment. Since the near-isogenic non-transgenic maize line ZD958 lacks glyphosate resistance, it would have been killed by glyphosate treatment. Commercially formulated isopropylamine salt of glyphosate was used at a concentration of 300 g a.e. L^−1^ (Roundup; Bayer, Leverkusen, Germany) to treat maize GG2 with the medium recommended dose of 900 g a.e. ha^–1^, according to the manufacturer’s manual. Maize was cultivated in accordance with regular agronomic practices. Samples were taken when the plants were at the preplant stage (PS), seedling stage with three leaves (SSV3), seedling stage with five leaves (SSV5), heading stage (HS), silking stage (SiS), dough stage (DS), and post-harvest stage (PHS). The five point sampling method was used to collect each soil sample. The rhizosphere soil of five plants from the same plot was shaken off and combined as a single sample to ensure the representativeness of the soil. The soil samples were stored at − 80 °C for DNA extraction.

### Physical and chemical characteristics of soil samples from ZD958 and GG2 at different developmental stages

The five indices of the physical and chemical characteristics of the soil, alkali-hydro nitrogen (AN), available potassium (AK), available phosphorus (AP), organic matter (OM), and pH values, were measured for all collected soil samples in accordance with the agricultural industry standard of the People’s Republic of China. AN was determined using the alkaline hydrolysis diffusion method; AK was analyzed using a flame photometer; AP was determined using the Olsen method; soil OM was determined using the oil bath heating potassium dichromate oxidation volumetric method; pH values were measured using the potentiometric method.

### Total DNA extraction, amplification, and illumina NovaSeq sequencing

Total microbial genomic DNA was extracted from the soil samples using a FastDNA SPIN Kit for Soil (Menlo Park, CA, US) according to the manufacturer’s instructions. The V3V4 region of the 16S rRNA gene from bacteria and the internal transcribed spacer (ITS) of the rRNA genes from fungi were amplified using the primer pairs 338F-806R and ITS1F-ITS2, respectively, which are listed in Table S6. A TruSeq^®^ DNA PCR-Free Sample Preparation Kit (Illumina, DE, USA) was used for library construction. Library quality was assessed using a QUBIT fluorometer and by qPCR, and the libraries were sequenced on the Illumina NovaSeq sequencing platform by Novogene Bioinformatics Technology Co., Ltd. (Beijing, China).

### Sequence processing and bioinformatics analysis

The assembled tags were clustered into operational taxonomic units (OTUs) at 97% similarity level using the UPARSE algorithm in USEARCH version 7.0.1090 (Edgar 2013). Representative OTU sequences were taxonomically classified using Ribosomal Database Project (RDP) Classifier v.2.2 with a minimal 50% confidence estimate in reference to SSUrRNA database for bacteria and fungi using the Mothur and SILVA138 methods (Wang et al. 2007; Quast et al. 2013). Based on the taxonomical classification and the Bray–Curtis distances of all samples, a series of bacterial and fungal community-related analyses was then performed. The diversity (Shannon, Simpson and PD_whole_tree indices) and richness (OTUs, Chao1, and ACE indices) of the samples were determined using QIIME software. A Venn diagram was produced to show the unique and shared OTUs present in the bacterial and fungal communities in rhizosphere soils. Principal Coordinates Analysis (PCoA) was also conducted using vegan software to determine the differences and similarities among the bacterial and fungal communities in rhizosphere soils associated with growth stages or maize lines. Furthermore, NMPANOVA (nonparametric multivariate analysis, also known as PERMANOVA or Adonis) and variance partitioning analysis (VPA) were conducted using vegan software to further confirm significant associations between two or more groups, including the maize lines and growth stages. Canonical Correlation Analysis (CCA) and Spearman correlation analysis were performed using the envfit and bioenv functions, respectively, to calculate the impact of each environmental factor on species distribution. Finally, LEfSe was conducted to support the high-dimensional taxonomical comparisons using LEfSe software.

## Supplementary Information

Below is the link to the electronic supplementary material.

Supplementary file1 (PDF 724 KB)

Supplementary file2 (PDF 738 KB)

Supplementary file3 (PDF 4284 KB)

Supplementary file4 (DOCX 13 KB)

Supplementary file5 (XLSX 12 KB)

Supplementary file6 (XLSX 10 KB)

Supplementary file7 (XLSX 11 KB)

Supplementary file8 (XLSX 11 KB)

Supplementary file9 (XLSX 12 KB)

Supplementary file10 (XLSX 9 KB)

## Data Availability

The raw sequence data have been deposited in Genome Sequence Archive in the National Genomic Data Center (GSA Accession No. CRA019197 for 16S and CRA019198 for ITS).
