# Supplementary figures and images for: Effects of cultivating biotech maize GG2 and glyphosate treatment on the rhizospheric microbial community structure
Source: aBIOTECH. 2025 Mar 12;6(2):174–88. doi: 10.1007/s42994-025-00205-8 (PMC12237839; doi:10.1007/s42994-025-00205-8)

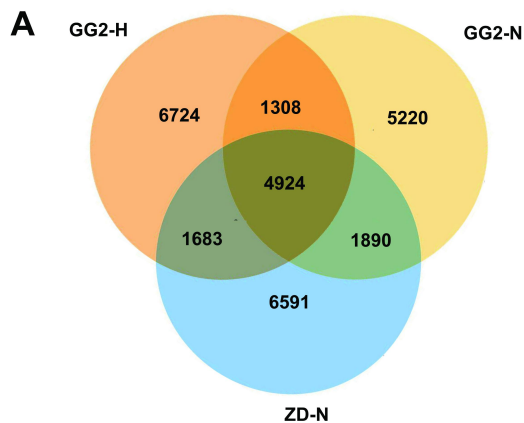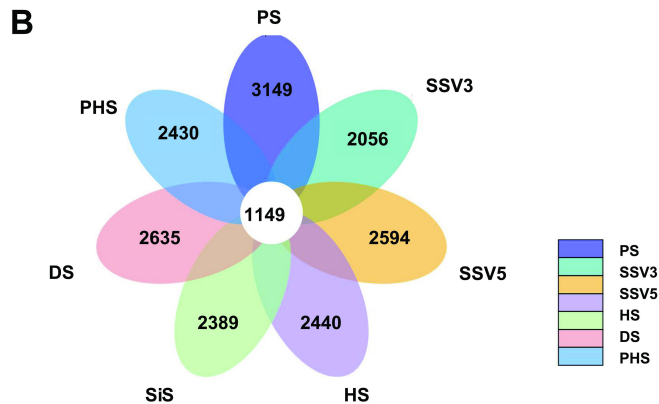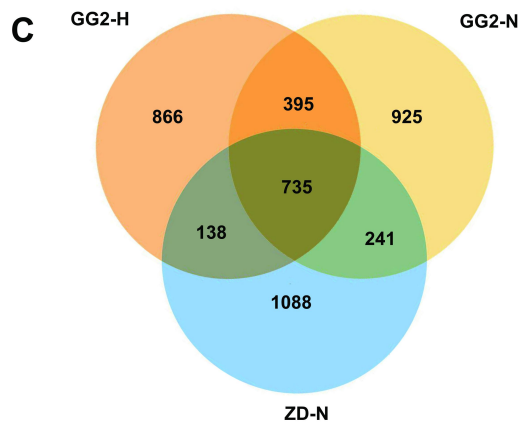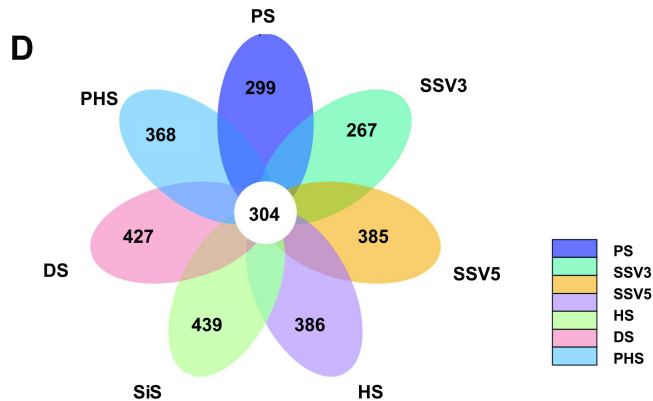

Supplement: Supplementary file 1 — Supplementary file1 (PDF 724 KB) [file 42994_2025_205_MOESM1_ESM.pdf]
