# Supplementary material for: Effects of cultivating biotech maize GG2 and glyphosate treatment on the rhizospheric microbial community structure
Source: aBIOTECH. 2025 Mar 12;6(2):174–88. doi: 10.1007/s42994-025-00205-8 (PMC12237839; doi:10.1007/s42994-025-00205-8)
Supplement: Supplementary file 2 — Supplementary file2 (PDF 738 KB) [file 42994_2025_205_MOESM2_ESM.pdf]

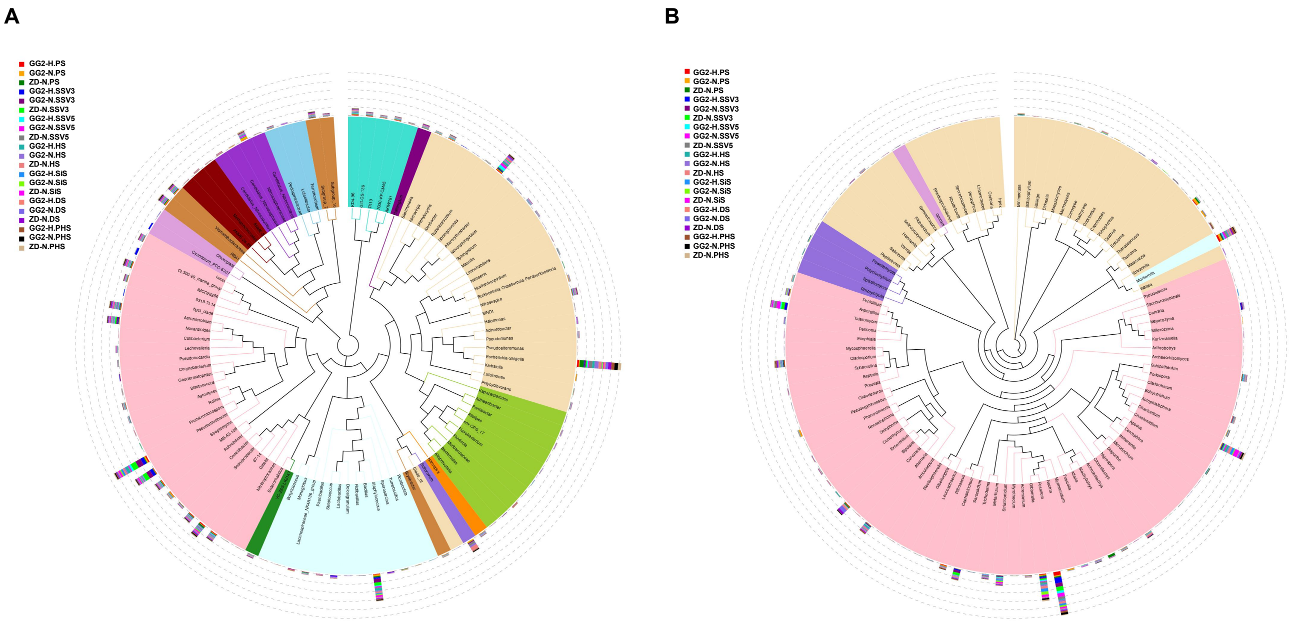
A
B
GG2-H.PS
GG2-N.PS
ZD-N.PS
GG2-H.SSV3
GG2-N.SSV3
ZD-N.SSV3
GG2-H.SSV5
GG2-N.SSV5
ZD-N.SSV5
GG2-H.HS
GG2-N.HS
ZD-N.HS
GG2-H.SiS
GG2-N.SiS
ZD-N.SiS
GG2-H.DS
GG2-N.DS
ZD-N.DS
GG2-H.PHS
GG2-N.PHS
ZD-N.PHS
