# Supplementary material for: Effects of cultivating biotech maize GG2 and glyphosate treatment on the rhizospheric microbial community structure
Source: aBIOTECH. 2025 Mar 12;6(2):174–88. doi: 10.1007/s42994-025-00205-8 (PMC12237839; doi:10.1007/s42994-025-00205-8)
Supplement: Supplementary file 4 — Supplementary file4 (DOCX 13 KB) [file 42994_2025_205_MOESM4_ESM.docx]

**Supplementary Figure legends:**

**Fig. S1 Composition of the bacterial and fungal communities in the rhizosphere soils of GG2 and ZD958.** Venn diagram of bacteria (**A**) and fungi (**C**) showing variable overlaps among GG2-H, GG2-N, and ZD-N; Venn diagram of bacteria (**B**) and fungi (**D**) showing variable overlaps among seven stages of plant growth. The numbers in circles represent the number of operational taxonomic units (OTUs). PS: preplant stage. GG2-H: GG2 treated with glyphosate at the seedling stage with four leaves. GG2-N: GG2 without glyphosate treatment. ZD-N: ZD958 without glyphosate treatment. PH: preplant stage. SSv3: seedling stage with three leaves. SSv5: seedling stage with five leaves. HS: heading stage. SiS: silking stage. DS: dough stage. PHS: post-harvest stage.

**Fig. S2 Phylogenetic classification of bacteria and fungi in the rhizosphere. A** Phylogenetic classification based on the V3V4 hypervariable sequences of the 16S rRNA gene for the top 100 bacterial genera for all samples. **B** Phylogenetic classification based on the ITS of the 18S rRNA gene and hypervariable sequences of the 5.8S rRNA gene for the top 100 fungal genera for all samples.

**Fig. S3 Linear discriminant analysis effect size (LEFSE) analysis of biomarkers of the bacterial and fungal communities in the rhizosphere soils of ZD958, which served as the control for HGK60 in 2021 and GG2 in 2022. A** The composition of bacteria in the rhizosphere soil related to seasons in the rhizosphere soils of ZD958, which served as the control for HGK60 in 2021. **B** The composition of fungi in the rhizosphere soil related to seasons in the rhizosphere soils of ZD958, which served as the control for HGK60 in 2021. **C** The composition of bacteria in the rhizosphere soil related to seasons in the rhizosphere soils of ZD958, which served as the control for GG2 in 2022. **D** The composition of fungi in the rhizosphere soil related to seasons in the rhizosphere soils of ZD958, which served as the control for GG2 in 2022.
