# Supplementary material for: Effects of cultivating biotech maize GG2 and glyphosate treatment on the rhizospheric microbial community structure
Source: aBIOTECH. 2025 Mar 12;6(2):174–88. doi: 10.1007/s42994-025-00205-8 (PMC12237839; doi:10.1007/s42994-025-00205-8)
Supplement: Supplementary file 3 — Supplementary file3 (PDF 4284 KB) [file 42994_2025_205_MOESM3_ESM.pdf]

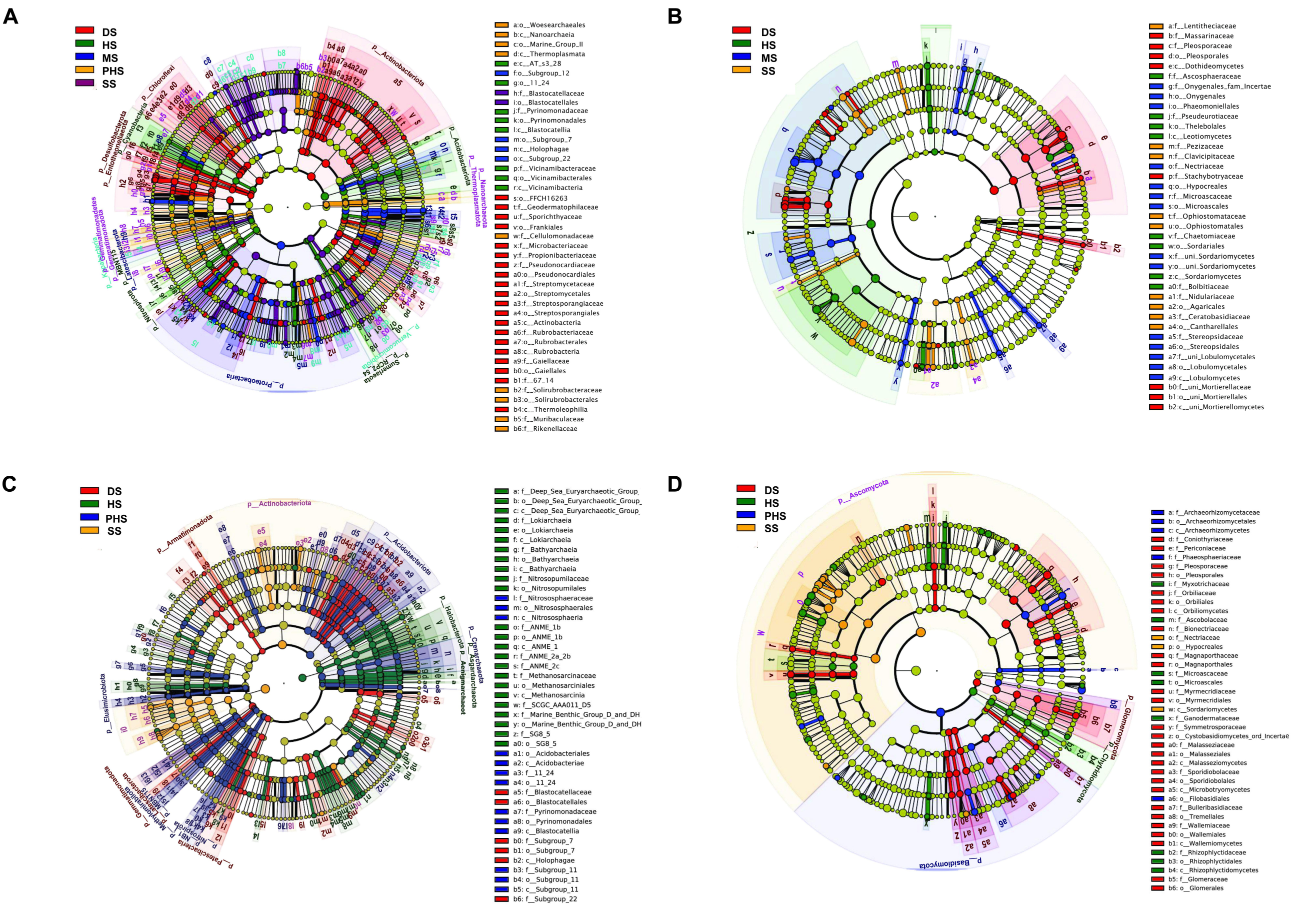

A
DS
HS
MS
PHS
SS
a:o__Woesearchaeales
b:c__Nanoarchaeia
c:o__Marine_Group_II
d:c__Thermoplasmata
e:c__AT_s3_28
f:o__Subgroup_12
g:o__11_24
h:f__Blastocatellaceae
i:o__Blastocatellales
j:f__Pyrinomonadaceae
k:o__Pyrinomonadales
l:c__Blastocatellia
m:o__Subgroup_7
n:c__Holophagae
o:c__Subgroup_22
p:f__Vicinamibacteraceae
q:o__Vicinamibacterales
r:c__Vicinamibacteria
s:o__FFCH16263
t:f__Geodermatophilaceae
u:f__Sporichthyaceae
v:o__Frankiales
w:f__Cellulomonadaceae
x:f__Microbacteriaceae
y:f__Propionibacteriaceae
z:f__Pseudonocardiaceae
a0:o__Pseudonocardiales
a1:f__Streptomycetaceae
a2:o__Streptomycetales
a3:f__Streptosporangiaceae
a4:o__Streptosporangiales
a5:c__Actinobacteria
a6:f__Rubrobacteriaceae
a7:o__Rubrobacterales
a8:c__Rubrobacteria
a9:f__Gaiellaceae
b0:o__Gaiellales
b1:f__67_14
b2:f__Solirubrobacteraceae
b3:o__Solirubrobacterales
b4:c__Thermoleophilia
b5:f__Muribaculaceae
b6:f__Rikenellaceae
p__Actinobacteriota
p__Acidobacteriota
p__Thermoplasmatota
p__Nanoarchaeota
p__Chloroflexi
p__Cyanobacteria
p__Desulfobacterota
p__Entotheonellaeota
p__Gemmatimonadota
p__Nitrospirota
p__Proteobacteria
p__Sumerlaeota
p__Verrucomicrobiota
B
DS
HS
MS
SS
a:f__Lentitheciaceae
b:f__Massarinaceae
c:f__Pleosporaceae
d:o__Pleosporales
e:c__Dothideomycetes
f:f__Ascosphaeraceae
g:f__Onygenales_fam_Incertae
h:o__Onygenales
i:o__Phaeomoniellales
j:f__Pseudeurotiaceae
k:o__Thelebolales
l:c__Leotiomycetes
m:f__Pezizaceae
n:f__Clavicipitaceae
o:f__Nectriaceae
p:f__Stachybotryaceae
q:o__Hypocreales
r:f__Microascaceae
s:o__Microascales
t:f__Ophiostomataceae
u:o__Ophiostomatales
v:f__Chaetomiaceae
w:o__Sordariales
x:f__uni_Sordariomycetes
y:o__uni_Sordariomycetes
z:c__Sordariomycetes
a0:f__Bolbitiaceae
a1:f__Nidulariaceae
a2:o__Agaricales
a3:f__Ceratobasidiaceae
a4:o__Cantharellales
a5:f__Stereopsidaceae
a6:o__Stereopsidales
a7:f__uni_Lobulomycetales
a8:o__Lobulomycetales
a9:c__Lobulomycetes
b0:f__uni_Mortierellaceae
b1:o__uni_Mortierellales
b2:c__uni_Mortierellomycetes
C
DS
HS
PHS
SS
a: f__Deep_Sea_Euryarchaeotic_Group_
b: o__Deep_Sea_Euryarchaeotic_Group_
c: c__Deep_Sea_Euryarchaeotic_Group_
d: f__Lokiarchaeia
e: o__Lokiarchaeia
f: c__Lokiarchaeia
g: f__Bathyarchaeia
h: o__Bathyarchaeia
i: c__Bathyarchaeia
j: f__Nitrosopumilaceae
k: o__Nitrosopumilales
l: f__Nitrososphaeraceae
m: o__Nitrososphaerales
n: c__Nitrososphaeria
o: f__ANME_1b
p: o__ANME_1b
q: c__ANME_1
r: f__ANME_2a_2b
s: f__ANME_2c
t: f__Methanosarcinaceae
u: o__Methanosarciniales
v: c__Methanosarcinia
w: f__SCGC_AAA011_D5
x: f__Marine_Benthic_Group_D_and_DH
y: o__Marine_Benthic_Group_D_and_DH
z: f__SG8_5
a0: o__SG8_5
a1: o__Acidobacteriales
a2: c__Acidobacteriae
a3: f__11_24
a4: o__11_24
a5: f__Blastocatellaceae
a6: o__Blastocatellales
a7: f__Pyrinomonadaceae
a8: o__Pyrinomonadales
a9: c__Blastocatellia
b0: f__Subgroup_7
b1: o__Subgroup_7
b2: c__Holophagae
b3: f__Subgroup_11
b4: o__Subgroup_11
b5: c__Subgroup_11
b6: f__Subgroup_22
p__Actinobacteriota
p__Armatimonadota
p__Acidobacteriota
p__Halobacterota
p__Crenarchaeota
p__Asgardarchaeota
p__Aenigmarchaeota
p__Elusimicrobiota
p__Gemmatimonadota
p__Methylomirabilota
p__Nitrospirota
p__Patescibacteria
D
DS
HS
PHS
SS
a: f__Archaeorhizomycetaceae
b: o__Archaeorhizomycetales
c: c__Archaeorhizomycetes
d: f__Coniothyriaceae
e: f__Periconiaceae
f: f__Phaeosphaeriaceae
g: f__Pleosporaceae
h: o__Pleosporales
i: f__Myxotrichaceae
j: f__Orbiliaceae
k: o__Orbiliales
l: c__Orbiliomycetes
m: f__Ascobolaceae
n: f__Bionectriaceae
o: f__Nectriaceae
p: o__Hypocreales
q: f__Magnaporthaceae
r: o__Magnaporthales
s: f__Microascaceae
t: o__Microascales
u: f__Myrmecridiaceae
v: o__Myrmecridiales
w: c__Sordariomycetes
x: f__Ganodermataceae
y: f__Symmetrosporaceae
z: o__Cystobasidiomycetes_ord_Incertae
a0: f__Malasseziaceae
a1: o__Malasseziales
a2: c__Malasseziomycetes
a3: f__Sporidiobolaceae
a4: o__Sporidiobolales
a5: c__Microbotryomycetes
a6: o__Filobasidiales
a7: f__Bulleribasidiaceae
a8: o__Tremellales
a9: f__Wallemiaceae
b0: o__Wallemiales
b1: c__Wallemiomycetes
b2: f__Rhizophlyctidaceae
b3: o__Rhizophlyctidales
b4: c__Rhizophlyctidomycetes
b5: f__Glomeraceae
b6: o__Glomerales
p__Ascomycota
p__Basidiomycota
p__Glomeromycota
p__Chytridiomycota
